# Central but not systemic administration of XPro1595 is therapeutic following moderate spinal cord injury in mice

**DOI:** 10.1186/s12974-014-0159-6

**Published:** 2014-09-10

**Authors:** Hans G Novrup, Valerie Bracchi-Ricard, Ditte G Ellman, Jerome Ricard, Anjana Jain, Erik Runko, Lise Lyck, Minna Yli-Karjanmaa, David E Szymkowski, Damien D Pearse, Kate L Lambertsen, John R Bethea

**Affiliations:** Department of Neurological Surgery, The Miami Project to Cure Paralysis, University of Miami Miller School of Medicine, 1095 NW 14th Ter R-48, Miami, FL 33136 USA; Department of Neurobiology Research, Institute of Molecular Medicine, University of Southern Denmark, Odense, J.B. Winsloewsvej 21 St, 5000 Odense C, Denmark; Coloplast A/S, Holtedam 1, 3050 Humlebæk Denmark, Denmark; Xencor Inc., 111 W Lemon Ave, Monrovia, CA 91016 USA; Department of Biomedical Engineering, Worcester Polytechnic Institute, 100 Institute Road, Worcester, MA 01609-2280 USA; Department of Biology, Drexel University, 3245 Chestnut St., PISB 123, Philadelphia, PA 19104 USA

**Keywords:** Functional outcome, Spinal cord injury, TLR4, TNFR2, Tumor necrosis factor

## Abstract

**Background:**

Glial cell activation and overproduction of inflammatory mediators in the central nervous system (CNS) have been implicated in acute traumatic injuries to the CNS, including spinal cord injury (SCI). Elevated levels of the proinflammatory cytokine tumor necrosis factor (TNF), which exists in both a soluble (sol) and a transmembrane (tm) form, have been found in the lesioned cord early after injury. The contribution of solTNF versus tmTNF to the development of the lesion is, however, still unclear.

**Methods:**

We tested the effect of systemically or centrally blocking solTNF alone, using XPro1595, versus using the drug etanercept to block both solTNF and tmTNF compared to a placebo vehicle following moderate SCI in mice. Functional outcomes were evaluated using the Basso Mouse Scale, rung walk test, and thermal hyperalgesia analysis. The inflammatory response in the lesioned cord was investigated using immunohistochemistry and western blotting analyses.

**Results:**

We found that peripheral administration of anti-TNF therapies had no discernable effect on locomotor performances after SCI. In contrast, central administration of XPro1595 resulted in improved locomotor function, decreased anxiety-related behavior, and reduced damage to the lesioned spinal cord, whereas central administration of etanercept had no therapeutic effects. Improvements in XPro1595-treated mice were accompanied by increases in Toll-like receptor 4 and TNF receptor 2 (TNFR2) protein levels and changes in Iba1 protein expression in microglia/macrophages 7 and 28 days after SCI.

**Conclusions:**

These studies suggest that, by selectively blocking solTNF, XPro1595 is neuroprotective when applied directly to the lesioned cord. This protection may be mediated via alteration of the inflammatory environment without suppression of the neuroprotective effects of tmTNF signaling through TNFR2.

## Background

Spinal cord injury (SCI) is a devastating clinical condition often resulting in paralysis below the level of injury and the development of secondary complications such as chronic pain and autonomic dysreflexia [1]. Broadly speaking, the primary injury is mediated by an initial traumatic event to the cord resulting in neuronal and glial injury/death and disruption of the blood-spinal cord barrier at the epicenter, which is almost immediately followed by a secondary wave of neuronal and gliatoxic sequelae including a rapid increase in glutamate, immunoregulatory cytokines such as tumor necrosis factor (TNF), toxic lipid metabolites, and, over time, the infiltration of peripheral blood leukocytes such as neutrophils, macrophages, and T-cells [2]. The immune system plays a dual role in both pathological destruction of neuronal tissue as well as in tissue repair and to some extent functional recovery [3-11]. Understanding the dichotomy between tissue destruction and tissue repair is essential for the development of effective therapies for SCI and other neurodegenerative disorders.

TNF is a pleiotropic cytokine important in the regulation of numerous physiological and pathological processes such as inflammation, autoimmunity, neurodegeneration, neuroprotection, demyelination, and remyelination [9,12-18]. There are two active forms of TNF, soluble-TNF (solTNF) and transmembrane-TNF (tmTNF), whose biological responses are primarily mediated by two distinct receptors, TNFR1 and TNFR2, respectively. TNFR1 has a death domain and signaling through this receptor has been implicated in both neuronal and oligodendrocyte death [9,15,17,19], whereas signaling through TNFR2 has been implicated in neuroprotection and remyelination [9,20,21]. Expression studies demonstrate that TNF is upregulated in the spinal cord within minutes to hours following injury and coincides with elevated glutamate, suggesting that injury-induced cytotoxicity may be mediated through additive or synergistic interactions between these and other soluble factors [2,10]. Studies using genetically engineered mice lacking either TNFR1 or TNFR2 show that TNF and its receptors indeed play a role in functional recovery and pathology following SCI [22]. While the behavioral data are difficult to interpret because the Basso, Beattie, and Bresnahan test was applied to evaluate changes in locomotor function in mice; the histological data are clear, deleting TNFR1 significantly reduces tissue damage [22]. In support of these studies, mice lacking TNFR1 (TNFR1^−/−^), the receptor preferentially activated by solTNF, are protected from experimental allergic encephalomyelitis (EAE) – a mouse model of multiple sclerosis – and have reduced pathology and normal remyelination [21,23,24]. Conversely, mice lacking TNF (TNF^−/−^) or TNFR2 (TNFR2^−/−^), the primary receptor for tmTNF, have worse functional outcome and do not remyelinate when exposed to EAE. Studies using genetically modified mice expressing only tmTNF show that this form of the cytokine, signaling through TNFR2, is sufficient to suppress the induction and progression of EAE [25]. Finally, we and others recently determined that systemic delivery of a selective inhibitor of solTNF, XPro1595, which binds solTNF forming inactive heterodimers [26], significantly improves functional recovery, reduces axonal damage, and promotes remyelination. In contrast, inhibition of solTNF and tmTNF with the non-specific TNF inhibitor, etanercept (decoy TNFR2 which blocks solTNF, tmTNF, and lymphotoxin [27]), proved neither therapeutic nor neuroprotective in EAE [9,20].

Based upon these and other data, we sought to investigate whether a pharmacological inhibition of solTNF or total TNF was therapeutic following SCI. Our results suggest that selectively targeting solTNF directly in the cord may be a new therapeutic avenue for this clinically devastating condition.

## Materials and methods

### Mice

Adult, female C57BL/6 mice were purchased from Charles River (USA) and transferred to The Miami Project to Cure Paralysis, University of Miami Miller School of Medicine, Miami, FL, USA, or from Taconic Ltd. (Ry, Denmark) and transferred to the Biomedical Laboratory, University of Southern Denmark, Odense, Denmark. Mice were housed in virus/antigen-free environments under diurnal lightning conditions and allowed free access to food and water. All experiments were approved by the University of Miami Animal Care and Use Committee (#09-010) and the Danish Animal Inspectorate (2008/561-1526).

### Induction of spinal cord injury

Surgeries were performed at the Animal and Surgical Core Facility of the Miami Project to Cure Paralysis or at the Biomedical Laboratory, University of Southern Denmark.

Mice were anaesthetized using a ketamine (100 mg/kg, VEDCO Inc., Saint Joseph, MO, USA)/xylazine (10 mg/kg, VEDCO) cocktail, laminectomized between vertebrae T8 and T10, and the impactor lowered at a predetermined impact force resulting in an approximate displacement of 500 μm (moderate injury). Contusion injury was induced with the mouse Infinite Horizon-0400 SCI Contusion Device (Precision Systems and Instrumentation, LLC, Fairfax Station, VA, USA) [3].

Sham-lesioned mice were subjected to laminectomy without displacement of the spinal cord and otherwise treated exactly like SCI-lesioned mice.

For central administration, micro-osmotic pumps were administered (see below) and mice were sutured and injected s.c. with 1 mL lactated Ringer’s Fluid USP (B. Braun, L7502, Bethlehem, PA, USA) to prevent dehydration, and housed separately in a recovery room, where their post-surgical health status was observed during a 24 to 48 h recovery period. Thereafter, mice were observed twice daily for activity level, body temperature, respiratory rate, and general physical condition. Manual bladder expression was performed twice a day until bladder function was regained. Body weight was monitored weekly. In addition, mice received s.c. prophylactic injections of antibiotic gentamicin (40 mg/kg) for 7 days following SCI to prevent urinary tract infections.

### Drug treatment

Immediately after surgery, mice were implanted with a micro-osmotic pump (Alzet model 1003D, Durect Corporation, Cupertino, CA, USA), which, for a period of 3 days, continuously delivered epidurally either XPro1595 (2.5 mg/mL concentration/1 μL/h), etanercept (Enbrel, 2.5 mg/mL/1 μL/h), or saline control (0.9% physiological saline/1 μL/h). Treatment dose was determined based on previous publications [28,29]. Stainless steel cannulas (ALZET Brain Infusion Kit 3) were attached to extension tubes and linked to the Alzet pumps, which were installed in subcutaneous pockets on the lateral back of the mice. Methylcyano acrylate was used to affix the catheters to T10, and wounds were closed with sutures.

Groups consisted of mice surviving either 7 (n = 12–15/group), 28 (n = 4–6/group), or 35 days (n = 14/group). Separate groups of mice were subjected to sham surgery and were allowed to recover from surgery for the 3 days that the micro-osmotic pumps delivered saline (n = 3), XPro1595 (n = 3), or etanercept (n = 3).

In order to test the efficacy of an alternative administration route, in a separate experiment we injected groups of mice s.c. with saline (n = 12), 10 mg/kg XPro1595 (n =12), or 10 mg/kg etanercept (n = 12). Treatment dose was determined based on previous publications [9,20,30]. First injection was performed 30 min after SCI, followed by s.c. injections every 3 days for 8 weeks. Time points of administration were based on previous findings of significantly elevated levels of TNF in the spinal cord within the first hour after SCI [10,31].

### Behavioral analysis

#### Basso Mouse Scale

Functional recovery of function after SCI was determined by scoring of the locomotor hind limb performance in the open field using the Basso Mouse Scale (BMS) system, a 0 to 9 rating system designed specifically for mice [3,32]. Under observer blinded conditions, mice were evaluated over a 4-min period, 1 and 3 days after SCI and weekly thereafter. Only mice with a score below 2 on day 1 were included in the study. Before surgery, mice were handled and pre-trained in the open field to prevent fear and/or stress behaviors that could bias the locomotor assessment.

#### Catwalk

The Catwalk test was performed, as described previously, to assess gait and motor coordination of mice [33]. The Catwalk equipment and software were purchased from Noldus Information Technology (Leesburg, VA, USA). Briefly, the mice continuously walked along a glass floor 1 m in length. Two fluorescent lamps that ran along the bottom of the floor illuminated the points of contact between the paws and floor. A video camera located beneath the glass floor recorded the gait of the mice. Each animal performed three runs. The following parameters were analyzed using the purchased software: the stride length, the base of support, paw contact area, and the intensity (assigned by the software) of the paw print.

#### Gridwalk

The grid walk was performed to analyze fine motor control. The grid consisted of steel rods (2 mm in diameter) that were spaced either 5 or 10 mm apart for a total distance of 1 m. The mice continuously walked along the rods. The number of slips for the hind leg was counted. One run was considered as back and forth, allowing for counting of the number of slips by both hind legs. Each mouse performed three runs. The number of slips over the three runs was averaged. The behavioral test was performed once a week on each animal. The placement of the rods was changed each week to prevent the mice from performing on “memory”.

#### Rung walk

In order to test stepping, inter-limb coordination, and balance, mice were tested on the rung walk when they reached a BMS score of 5. The rung walk consisted of two plates of transparent polymer, approximately 110 cm × 20 cm, with a 2.5 cm space between them. Rungs with a 2 mm diameter were placed using a specific pattern according to Metz and Whishaw [34]. The apparatus was placed on two cages with the home cage at one end, making the mice automatically walk in that direction. To avoid stopping or turning during trials, animals were pre-trained five times prior to surgery with the final test serving as baseline. Following SCI, mice were tested at 3, 4, and 5 weeks using a handheld GoPro HD camera with 48 fps. Data were evaluated frame by frame using QuickTime. Left and right scores were calculated as follows: 6, complete miss; 5, touching rung, but sliding off and losing balance; 4, touch, miss but no loss of balance; 3, replacement, mouse placed paw on rung but quickly moves it; 2, recorrection, aims for a rung but changes direction; 1, anterior or posterior placement; 0, perfect step. The total number of mistakes was plotted for analysis as previously described by Metz and Whishaw [34].

#### Thermal hyperalgesia

Thermal hyperalgesia (hind paw withdrawal from a normally innocuous heat source) was tested with a Hargreave’s heat source as described in detail by Berrocal et al. [35] (systemic studies) or by using the Plantar Test apparatus (37370, Ugo Basile, Comerio VA, Italy) (central studies). For peripherally administered studies, the test was repeated on the opposite paw and repeated two more times. In between each measurement, the mouse was allowed to recover for 15 min. The latency times of three measurements per foot were averaged. For the centrally administrated studies, each paw was tested five times with at least 2 min break in between. The lowest and highest reflex latency scores of each paw were discarded and the bilateral mean was calculated and plotted. The behavioral test was performed once a week on each animal.

#### Open field

The open field test was performed with a non-transparent, squared plastic box (45 × 45 × 45 cm) over a period of 10 min [36]. Movements were tracked using the SMART video tracking software (Panlab, Barcelona, Spain) connected to a video camera (SSC-DC378P, Biosite, Stockholm, Sweden). The distance travelled (m), speed (cm/sec), and the entries into the three zones (wall, interperiphery, and center of the box) were recorded automatically. Rearing, grooming, urination, and droppings were recorded manually and are presented as number (n) of events.

### Tissue processing

#### Histopathology and immunohistochemistry

For paraffin histopathology and immunohistochemical analysis, mice were deeply anaesthetized using an overdose of pentobarbital (200 mg/mL) containing lidocaine (20 mg/mL) and perfused through the left ventricle with cold phosphate buffered saline (PBS) followed by 4% paraformaldehyde in PBS. The spinal cords were quickly removed and tissue segments containing the lesion area (1 cm centered on the lesion) were paraffin-embedded and cut into 10 parallel series of 15-μm thick microtome sections. Sections were stored at room temperature until further processing. For mice with 8 weeks survival and treated s.c. with anti-TNF, spinal cords were cryoprotected in 0.1 M PBS + 20% sucrose and cut into 10 series of 25-μm thick cryostat sections and stored at −80°C until further processing.

### Klüver-Barrera Luxol Fast Blue staining for myelinated fibers

For evaluation of lesion pathology, sections were stained in Luxol Fast Blue (0.1% Luxol Fast Blue in 95% EtOH and 0.05% acetic acid) at 60°C overnight. The next day, sections were rinsed in 96% EtOH and distilled H_2_O, immersed briefly in 0.05% Li_2_CO_3_ in distilled water, and differentiated in 70% EtOH. Next, sections were rinsed thoroughly in distilled H_2_O and immersed in 0.05% Li_2_CO_3_ to stop further differentiation. Sections were then placed in hematoxylin, rinsed in running tap water, and immersed briefly in eosin solution. Finally, sections were rinsed in 70% EtOH, followed by 95% and 99% EtOH, placed in 2 × xylene prior to mounting with Depex. Prior to staining, paraffin embedded sections were deparaffinized 3 × 3 min in xylene, 3 × 2 min in 99% EtOH, and 2 × 2 min in 96% EtOH.

### Determination of injury volume

The injury volume was determined from the area of every tenth section sampled by systematic uniform random sampling. The area of the lesion site was estimated essentially as described by Bethea et al. [10].

Digital images were acquired using the 4× lens on an Olympus BX51 microscope fitted with an Olympus DP70 digital camera and a computerized specimen stage (Prior, Multicontrol 2,000 MW). The setup was connected to a PC running Visiopharm Integrator Software (Visiopharm a/s, Hørsholm, Denmark) for image analysis. Calibrated digital images of the sampled sections were acquired and subjected to semi-automatic segmentation in VisioMorph (Visiopharm a/s) in a multistep protocol: 1) labelling of the entire tissue section based on thresholding (× <59) in the green channel with (5.5) media filtering; 2) labelling of all myelinated fibers based on thresholding (× <90) in a channel created by subtracting the red channel information from the blue channel information, followed by (5.5) media filtering; 3) post-processing to remove small objects; 4) outlining of the lesion area by drawing; 5) outlining of intact grey matter by drawing; 6) automated calculation of the areas belonging to A) lesion, B) intact grey matter, C) myelinated fibers, and D) total section area. These areas were summarized and multiplied by the section distance, resulting in an estimate of the total volumes after dehydration and paraffin embedding.

### Western blotting

Spinal cords of mice used for western blotting included the following groups: naive mice (n = 3), saline-treated SCI mice with 7 days survival (n = 3) and 28 days survival (n = 3), XPro1595-treated SCI mice with 7 days survival (n = 3) and 28 days survival (n = 4), and etanercept-treated SCI mice with 7 days survival (n = 4) and 28 days survival (n = 5). In total, 1.5 cm of spinal cord tissue centered on the injury was quickly removed, flash-frozen in liquid nitrogen, and stored in RNAse-free Eppendorf tubes at −80°C until further processing.

### Protein extraction

Proteins were extracted as previously described [3]. Briefly, samples were homogenized in RIPA buffer (0.01 M sodium phosphate pH 7.2, 0.15 M NaCl, 1% NP40, 1% sodium deoxycholate, 0.1% SDS, 2 mM EDTA) supplemented with Roche complete protease inhibitor cocktail, mixed end-over-end at 4°C for 30 minutes, and centrifuged at 14,000 rpm for 10 min at 4°C. The supernatants were transferred to fresh tubes and stored at −80°C.

### Protein quantification

Protein quantification was performed using *DC* Protein Assay (Bio-Rad, Hercules, CA, USA).

### Protein electrophoresis and transfer

Equal amounts of protein lysates were resolved by SDS-PAGE on 10 or 15% gels and transferred to nitrocellulose membrane (Bio-Rad).

### Protein visualization

Following blocking in 5% non-fat milk in tris buffered saline + Triton (TBS-T), membranes were probed overnight at 4°C with one of the following antibodies: recognizing glial fibrillary acidic protein (GFAP, 1:500, BD Pharmingen), growth associated protein 43 (GAP43, 1:5,000), ionized calcium binding adapter molecule 1 (Iba1, 1:400, Wako), myelin basic protein (MBP, 1:500, Millipore), toll-like receptor 4 (TLR4, 1:200, Santa Cruz), and tumor necrosis factor receptor 2 (TNFR2, 1:200, Santa Cruz). After extensive washes in TBS-T, membranes were incubated with horseradish peroxidase-conjugated secondary antibodies (1:2,000, GE Healthcare for anti-mouse and anti-rabbit, 1:1,000, Jackson ImmunoResearch for anti-rat) for 30 min at room temperature. Detection was performed with SuperSignal West Pico chemiluminescent substrate (Thermo Scientific). Quantification was performed using Quantity One software from Bio-Rad. Blots were normalized using either mouse anti-β-actin (1:500, Santa Cruz) or rabbit anti-β-actin (1:1,000, Cell Signaling).

### Immunostaining

Immunostaining for macrophage/microglia-specific Iba1 was performed on paraffin-embedded sections using rabbit anti-Iba1 (#019-19741, Wako) (1:600) essentially as described in Dissing-Olesen et al. [37]. Sections were counterstained with Toluidine blue. All sections were stained at the same time.

### Immunofluorescent staining

Immunofluorescent staining for GFAP was performed on paraffin-embedded sections using mouse anti-human GFAP-Alexa Fluor® 488 (A21282, Life Technologies) (1:400) essentially as described in Clausen et al. [38].

### Estimation of the total number of Iba1^+^ cells 7 days after SCI

Using an approximated stereological counting technique [3,39], we estimated the total number of Iba1^+^ cells in the spinal cord 7 days after injury in mice treated epidurally with either XPro1595 (n = 6), etanercept (n = 5), or saline (n = 4). Cells with a clearly identifiable Toluidine blue-stained nucleus in conjunction with a detectable immunohistochemical signal were counted on approximately 7 sections centered on the lesion site and separated by 150 μm from each animal, using a × 100 objective and a 2,470 μm^2^ frame area stepping 150 μm/150 μm in the XY-position using the New Computer Assisted Stereological Toolbox (NewCAST) from Visiopharm (Hoersholm, Denmark). The total number (N) of cells in each animal was estimated using the formula: Estimate of N = ∑Q ⋅ (1/ssf) ⋅ (1/asf) ⋅ (1/tsf), where 1/tsf is the thickness sampling fraction (1/tsf = 1), 1/ssf is the sampling section fraction (1/ssf = 10), and 1/asf is the area sampling fraction (22,500/2,470) as previously described [3,39]. Due to variations in length of the longitudinally cut spinal cord sections, total cell numbers are given as number of Iba1^+^ cells/mm^2^. An average of 17 mm^2^ was estimated from each animal.

### Densitometric analysis of Iba1^+^ cells 35 days after SCI

Due to the density of Iba1^+^ cells 35 days after SCI we were unable to systematically perform stereological estimations of the total number of Iba1^+^ cells at this time point. Therefore, we performed a densitometric analysis on spinal cord sections from mice treated epidurally with either saline (n = 4), XPro1595 (n = 5), or etanercept (n = 5) using Image J analysis software (NIH) as per directions of the Image J developers (http://rsb.info.nih.gov/ij) and as described before [40]. Photo documentation was done using an Olympus BX51 microscope with an Olympus DP73 camera connected to a PC setup with the Olympus CellSens software. All TIFF files were grayscaled (8 bits) using Adobe Photoshop CS5 for Mac, pictures imported as group pictures (17 sections per animal) into Image J, and background subtracted. Each section was delineated using the polygon selection tool and the densitometry measured across the section was estimated using the Log_10_(mean value/255) calculation.

### Data analysis

Comparisons were performed using repeated measures two-way ANOVA or one-way ANOVA followed by Tukey’s post-hoc test. Analyses were performed using Prism 4.0b software for Macintosh (GraphPad Software). Statistical significance was established for *P* <0.05.

## Results

### The effect of systemically administered anti-TNF therapy on functional recovery after SCI

**Figure 1:**
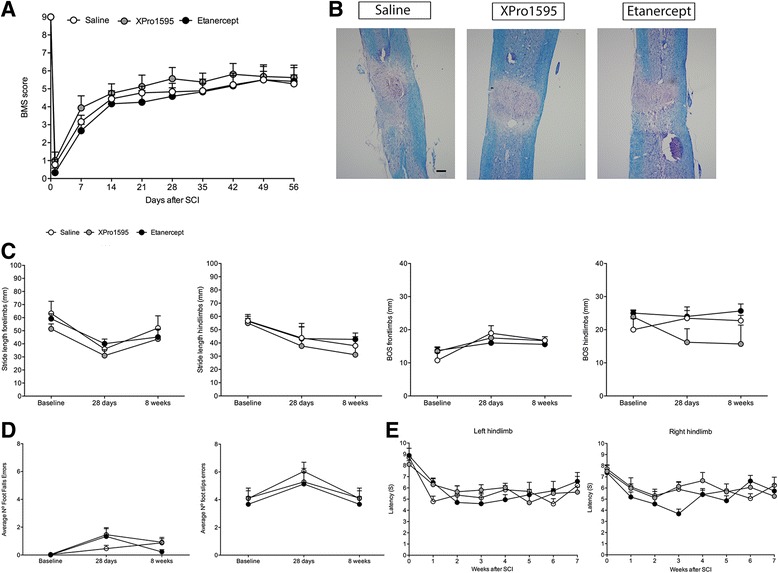
**Systemically administered anti-TNF therapy does not affect motosensory functions or lesion size after SCI. (A)** Analysis of Basso Mouse Scale (BMS) scores in mice treated systemically every third day with either XPro1595, etanercept, or saline for 8 weeks (56 days) showed comparable locomotor performance in the open field arena (*P* = 0.64), though all groups significantly improved their BMS score over time (****P <0.0001, two-way repeated masures ANOVA). **(B)** Luxol fast blue stained thoracic spinal cord sections from mice treated with either saline, XPro1595, or etanercept and allowed 8 weeks survival after SCI. Scale bar: 100 μm. **(C)** Catwalk analysis showing changes in front and hind limb stride length and front- and hind limb base of support (BOS) over time after SCI. No difference was observed between saline-, XPro1595-, and etanercept-treated mice; however, all mice displayed significant changes in stride length (**P <0.01 for hind limbs and *****P <0.0001 for front limbs, respectively) and BOS on the front limbs (**P <0.01) over time. **(D)** Gridwalk analysis showing changes in the average number of foot falls errors and average number of foot slips errors over time after SCI. No difference was observed between saline-, XPro1595-, and etanercept-treated mice; however, all mice displayed significant changes over time both in foot falls errors (***P <0.001) and foot fall slips (***P <0.001). **(E)** Analysis of thermal hyperalgesia after SCI showed no differences in latencies to remove left (*P* = 0.27) or right (*P* = 0.16) hind limbs after stimulation between saline-, XPro1595-, or etanercept-treated mice. All mice did, however, display significant changes over time in the latency to remove both the left and right hind limbs after stimulation (****P <0.0001).

Previous studies have demonstrated that TNF is upregulated in the cord following trauma or disease and that it participates in secondary injury mechanisms [2,10]. Using a mouse model of multiple sclerosis, we and others determined that XPro1595 was therapeutic and neuroprotective when administered systemically [9,20]. Therefore, based upon these earlier studies, we investigated whether systemically administered XPro1595 would improve functional recovery and reduce tissue damage in a mouse model of traumatic SCI. In this study, animals were randomly assigned to three different groups: saline (vehicle), XPro1595, or etanercept. These drugs were delivered s.c. within 30 min of injury and then again every 3 days for 8 weeks. To test whether subcutaneous administration of either XPro1595 or etanercept affected functional recovery after SCI, locomotor performance in the open field was recorded on day 1 and day 3 and then weekly for 8 weeks, and scored with the BMS. We determined there were no differences between any of the treatment groups (final BMS ± SEM: saline: 5.3 ± 0.5; XPro1595: 5.6 ± 0.6; and etanercept: 5.4 ± 0.9; *P* = 0.64; Figure 1A). Mice were also evaluated using catwalk analysis at 4 and 8 weeks after SCI (Figure 1C). All treatment groups displayed significant changes over time in stride length of the forelimbs (*P* <0.0001) and hind limbs (*P* <0.01), but no differences were observed between the treatments (*P* >0.05). Base of support also significantly changed over time for the forelimbs (*P* <0.01) but not for the hind limbs (*P* = 0.72) (Figure 1C). Grid walk analysis at 4 and 8 weeks after SCI also did not show any differences between treatment groups when investigating the average number of foot fall errors (*P* = 0.33) and the average number of foot slip errors (*P* = 0.74, Figure 1D); however, we did observe SCI-induced changes in both foot fall errors and foot slip errors over time (*P* <0.001 for both).

Histological evaluation of the injured cords 8 weeks after SCI (Figure 1B) did not reveal any differences in lesion size between groups. Importantly, we found no significant differences in total body weight after SCI between the different groups (*P* = 0.43, data not shown), though all three groups displayed significant changes in total body weight over time (*P* <0.0001).

### The effect of centrally administered anti-TNF therapy on functional recovery after SCI

**Figure 2:**
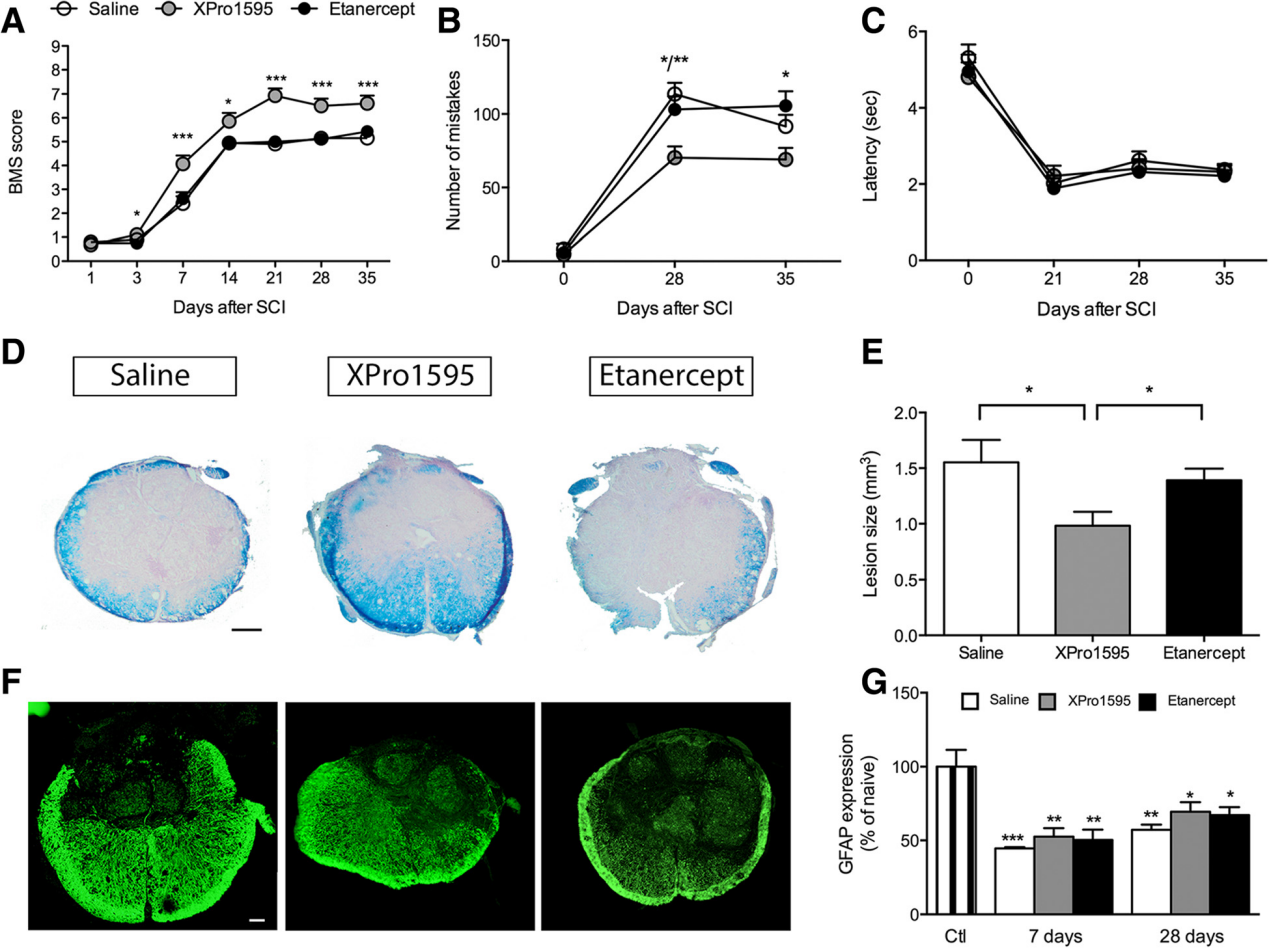
**Centrally administered XPro1595 improves motor functions and decreases lesion size after SCI. (A)** Analysis of BMS scores in mice treated centrally for three consecutive days with either saline, XPro1595, or etanercept showed that XPro1595-treated mice significantly improved their BMS score from 3 to 35 days after SCI compared to both saline- and etanercept-treated mice (*P <0.05 and ***P <0.001, two-way repeated measures ANOVA). All groups of mice significantly improved their BMS score over time (***P <0.001). **(B)** Rung walk analysis showed that XPro1595-treated mice significantly decreased their number of mistakes compared to saline- and etanercept-treated mice (*P <0.05 and **P <0.01). **(C)** Thermal stimulation using the Hargreave’s test showed no differences in latency time to withdraw paws between saline-, XPro1595-, and etanercept-treated mice. All mice decreased their latency to remove their paws over time after SCI (****P <0.0001). **(D)** Luxol fast blue stained thoracic spinal cord sections from mice treated with either saline, XPro1595, or etanercept and allowed 35 days survival after SCI. Scale bar: 100 μm. **(E)** Analysis of lesion volumes 35 days after SCI showed that the lesion size was significantly smaller in XPro1595-treated mice compared to both saline- and etanercept-treated mice (one-way ANOVA, followed by Tukey’s test). **(F)** Representative thoracic spinal cord sections from saline-, XPro1595-, and etanercept-treated mice stained for anti-GFAP allowed 35 days survival. Scale bar: 100 μm. **(G)** Quantification of GFAP protein expression in spinal cord tissue of saline-, XPro1595-, and etanercept-treated mice at 7 and 28 days after SCI. Data are normalized to β-actin protein expression. Results, expressed as percent of control, are the mean ± SEM of three animals per group. *P <0.05, **P <0.01, and ***P <0.001 versus control by one-way ANOVA with Tukey’s test.

These results were surprising because we and others had previously determined that the systemic administration of XPro1595 was therapeutic in a mouse model of multiple sclerosis [9,20]. Numerous studies have reported that TNF, mRNA, and protein, accumulate in the injured cord within hours of injury and persist for several weeks [10,41]. However, macrophages, the principal source of systemic TNF, do not accumulate in the injured cord in significant numbers for at least 72 h post trauma [10,42,43]. Based upon these data and the failure of systemically delivered XPro1595 to improve functional recovery, we reasoned that TNF produced within the injured cord by microglia, astrocytes, and other cell types may facilitate tissue destruction and that targeting this source of TNF may be required to promote functional recovery. To test this hypothesis, we delivered either XPro1595 or etanercept for 3 days directly to the lesion epicenter following SCI and quantified changes in motor performance. Using the BMS in the open field test, we determined that compared to saline or etanercept, XPro1595-treated mice exhibited a dramatic and significant improvement in functional recovery (Figure 2A). Our BMS data was supported by rung walk analysis (Figure 2B), where XPro1595-treated mice displayed significantly fewer mistakes compared to both saline- (*P* <0.01) and etanercept-treated (*P* <0.05) mice. In contrast, we found no differences in thermal hyperalgesia using the plantar test (Figure 2C), where all groups displayed similar paw withdrawal latency times after SCI. Latency times did, however, change over time after SCI in all three groups (*P* <0.0001). Histological analysis of the injured cords 35 days after SCI showed significantly smaller lesions in XPro1595-treated mice compared to both saline- and etanercept-treated mice (Figure 2D and E). Interestingly, there was no change in GFAP expression, indicating that inhibiting soluble TNF did not alter this one indicator of reactive astrogliosis (Figure 2F).

### Centrally administered XPro1595 improves general activity and reduces anxiety-like behaviors following SCI

**Figure 3:**
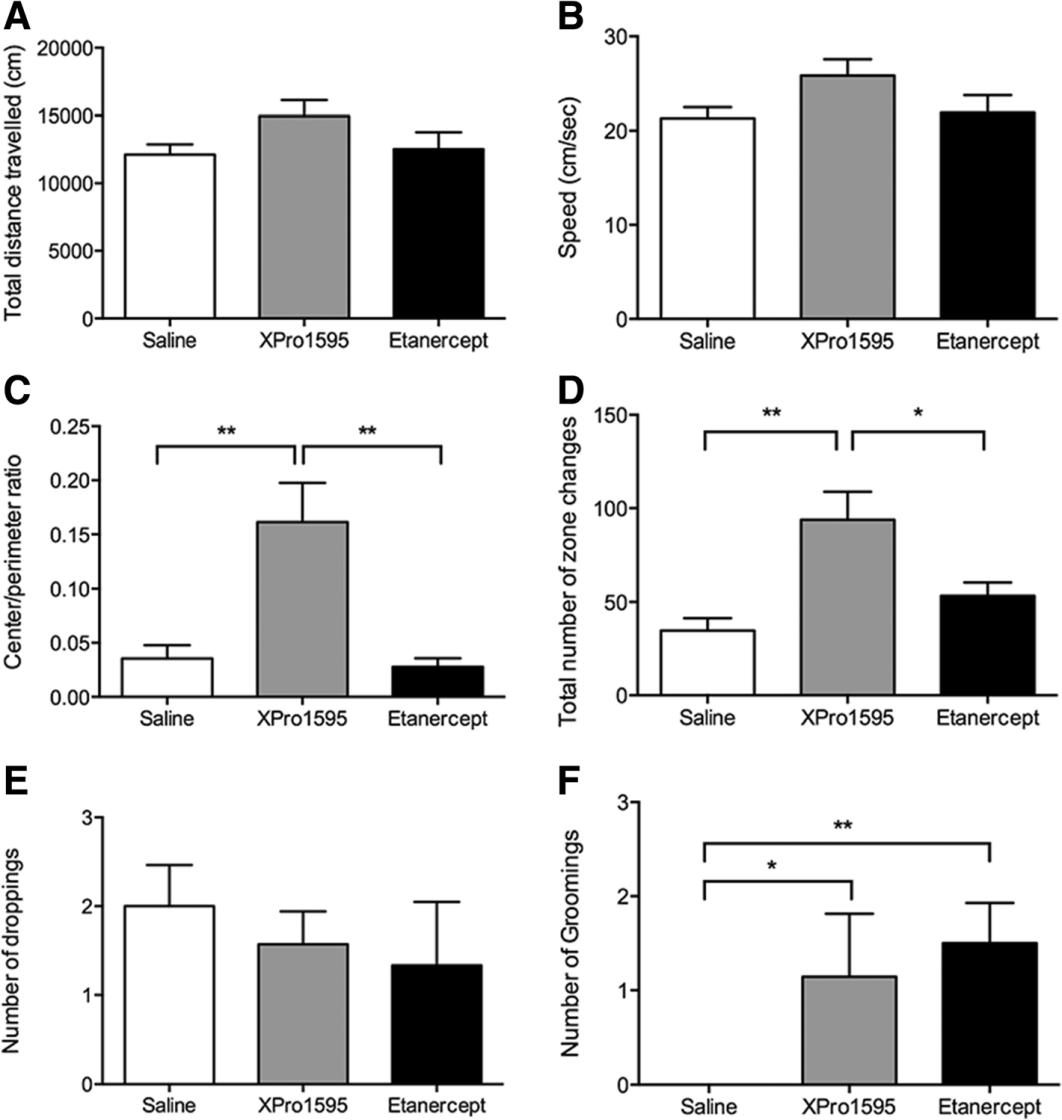
**Open field test analysis of SCI mice treated centrally with anti-TNF therapy for three consecutive days and allowed 35 days of survival after SCI. (A,B)** Analysis of locomotor activity in mice treated centrally with either saline, XPro1595, or etanercept and allowed 35 days survival after SCI showed that all mice travelled a similar distance **(A)** at comparable speeds **(B)** in the open field test. **(C,D)** Analysis of anxiety-related behavior in the open field test showed that XPro1595-treated mice displayed decreased anxiety-related behavior represented by increased center/perimeter ratio **(C)** and increased number of zone changes into the perimeter and center area **(D)**. **(E,F)** The number of droppings **(E)** was comparable in all groups of mice, whereas the number of groomings was increased both in XPro1595- and etanercept-treated mice compared to saline-treated mice. *P <0.05 and **P <0.01, one-way ANOVA followed by Tukey’s test.

We noticed that mice treated with XPro1595 directly to the cord, in general, looked healthier and exhibited more exploratory activity and movement when in their home cages. To address this formally, we performed open-field analysis of SCI mice treated with saline, XPro1595, or etanercept at 35 days post-injury, with observers blinded to the treatment groups, to measure changes in general activity and anxiety. We found no differences in the total distance travelled in the open field, nor in the speed at which the mice travelled, suggesting that XPro1595 or etanercept do not affect overall activity levels (Figure 3A and B). However, mice treated with XPro1595 spent significantly more time in the center of the testing area compared to both saline- and etanercept-treated mice (Figure 3C) and displayed significantly more zone changes (Figure 3D), suggesting less anxiety in XPro1595-treated mice. The number of droppings between groups was not different (Figure 3E). Both XPro1595- and etanercept-treated mice displayed an increased number of groomings compared to saline-treated mice (Figure 3F). Finally, most of the XPro1595-treated mice were capable of rearing at least once during testing, whereas none of the etanercept- or saline-treated mice were capable of rearing (data not shown).

### Anti-TNF therapy affects microglial/macrophage responses after SCI

**Figure 4:**
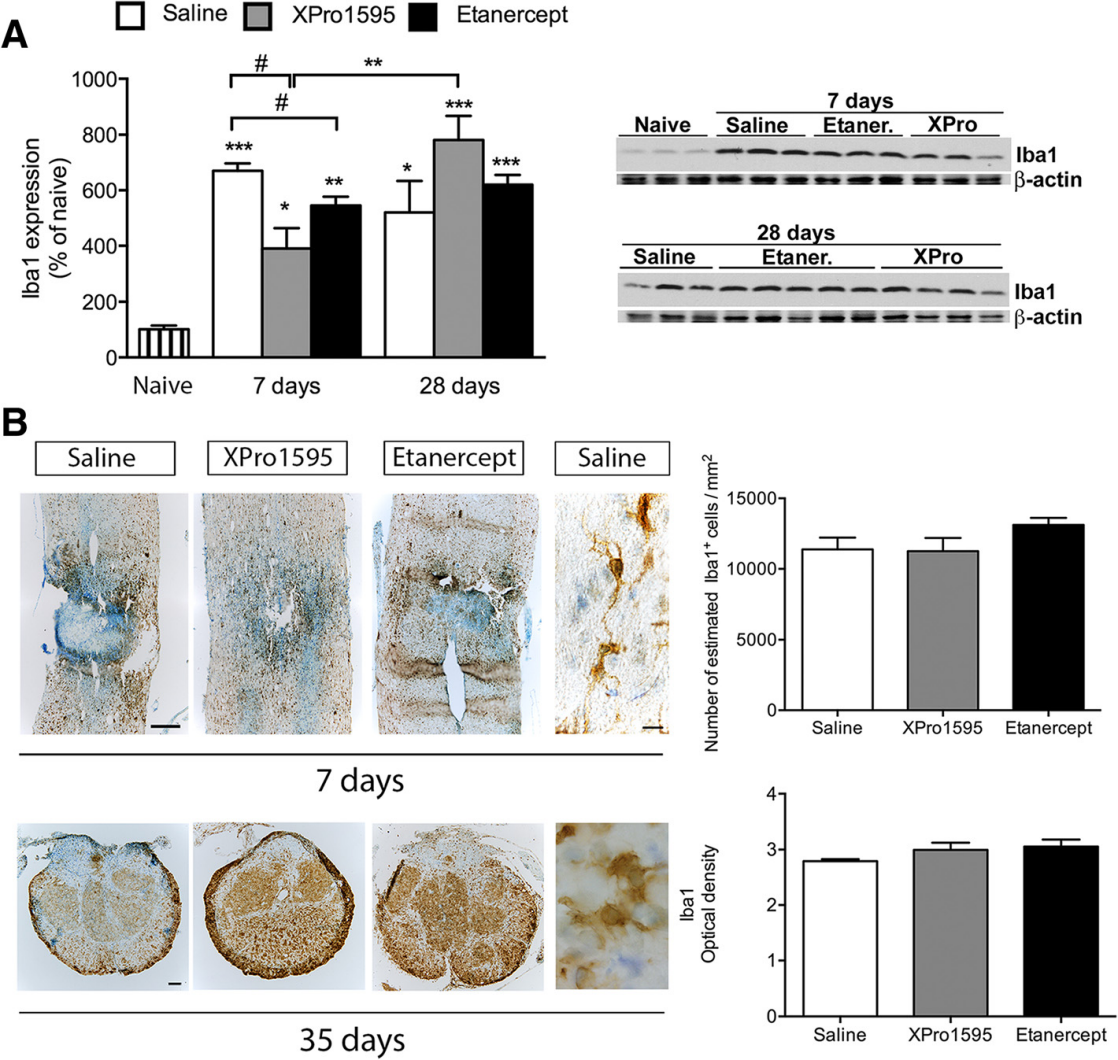
**Changes in Iba1 protein expression following central anti-TNF treatment after SCI. (A)** Quantification of Iba1 protein expression in spinal cord tissue of saline-, XPro1595-, and etanercept-treated mice at 7 and 28 days after SCI. Data are normalized to β-actin protein expression. Representative experiments are shown. Results, expressed as percent of control, are the mean ± SEM of three animals per group. #*P* <0.05 versus XPro1595 and etanercept; *P <0.05, **P <0.01, and ***P <0.001 versus control by one-way ANOVA with Tukey’s test. **(B)** Representative low magnification photomicrographs of thoracic spinal cord sections from saline-, XPro1595-, and etanercept-treated mice allowed 7 and 35 days survival after SCI and representative high magnification photomicrographs from saline-treated mice allowed 7 and 35 days survival and stained for anti-Iba1. Estimation of the total number of Iba1^+^ cells 7 days after SCI (upper graph) and densitometric analysis of Iba1^+^ cells 35 days after SCI (lower graph) displayed no difference in microglial/macrophage numbers between the different treatment groups. Scale bars: low magnification: 100 μm and high magnification: 10 μm.

Iba1 is often used as a marker to measure microglial and macrophage activation and accumulation following injury or disease to the central nervous system (CNS). Therefore, we investigated whether our treatment strategies regulated Iba1 expression and used this as a surrogate marker for cell activation and accumulation in the cord. Western blotting for Iba1 (Figure 4A) revealed a significant SCI-induced increase in Iba1 expression in all treatment groups both 7 days and 28 days after SCI, likely reflecting activation of resident microglia and infiltration of macrophages. At 7 days after SCI, Iba1 expression was more pronounced in saline-treated mice compared to both XPro1595- and etanercept-treated mice, suggesting that anti-TNF therapy decreases microglial activation and/or macrophage infiltration. However, this increase in Iba1 expression in saline-treated mice was not reflected in significantly increased numbers of Iba1^+^ cells in saline-treated mice (Figure 4B, upper graph), suggesting that microglial/macrophage activation was less pronounced in Xpro1595- and etanercept-treated mice. At 28 days, Iba1 expression remained elevated in all three groups compared to naive mice; in saline- and etanercept-treated mice to a similar extent as at 7 days, and with a further increase in XPro1595-treated mice (Figure 4A). This was also reflected in comparable Iba1 protein expression 35 days after SCI in all three groups of mice (Figure 4B, lower graph).

### XPro1595-treatment sustains MBP expression possibly through the upregulation of TNFR2 and TLR4 expression in the lesioned spinal cord

**Figure 5:**
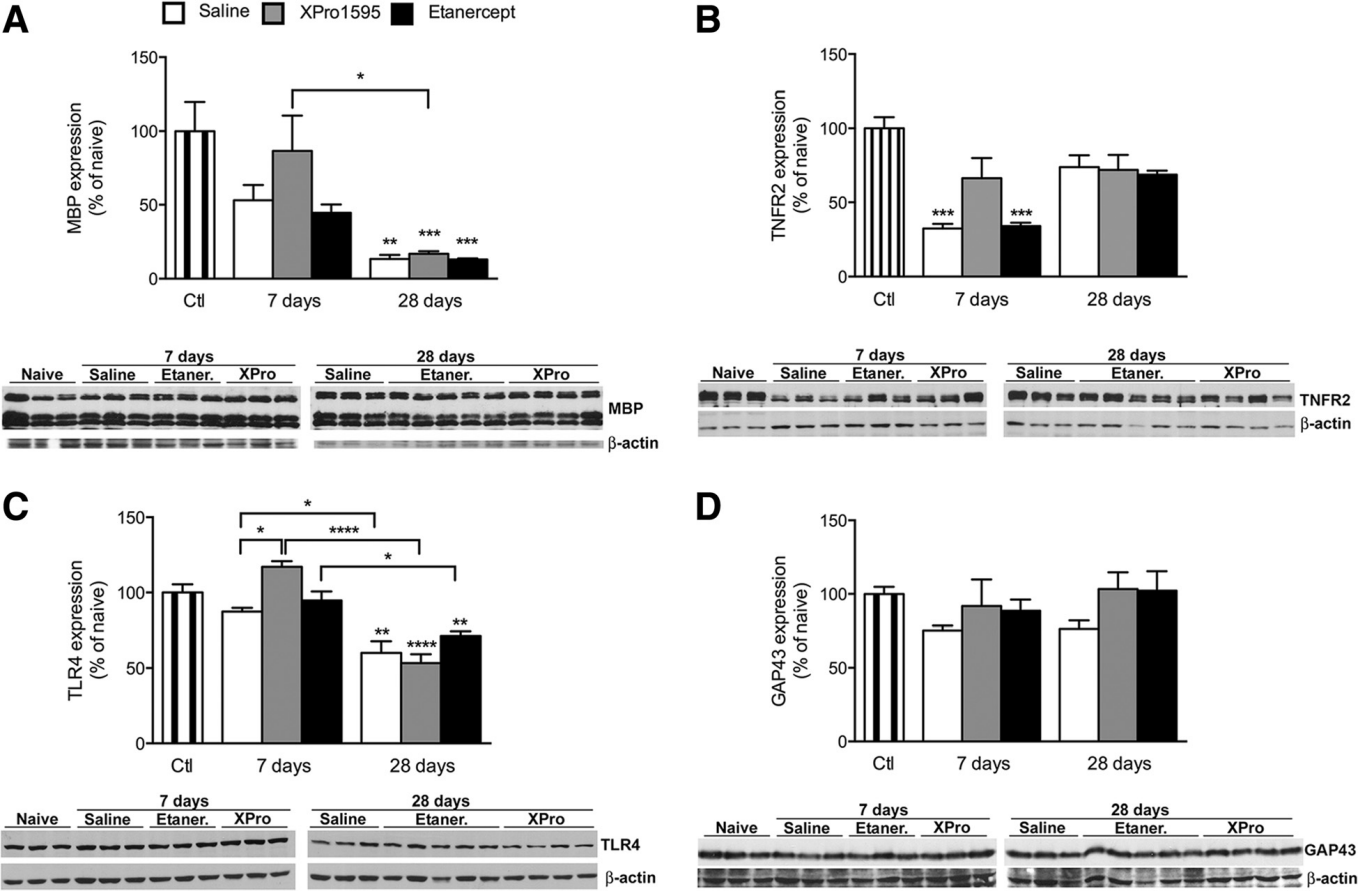
**Changes in MBP, TNFR2, TLR4, and GAP43 protein expression following central anti-TNF treatment after SCI. (A)** Quantification of MBP protein expression in spinal cord tissue of saline-, XPro1595-, and etanercept-treated mice at 7 and 28 days after SCI. **(B)** Quantification of TNFR2 protein expression in spinal cord tissue of saline-, XPro1595-, and etanercept-treated mice at 7 and 28 days after SCI. **(C)** Quantification of TLR2 protein expression in spinal cord tissue of saline-, XPro1595-, and etanercept-treated mice at 7 and 28 days after SCI. **(D)** Quantification of GAP43 protein expression in spinal cord tissue of saline-, XPro1595-, and etanercept-treated mice at 7 and 28 days after SCI. Data are normalized to β-actin protein expression. Representative experiments are shown. Results, expressed as percent of control, are the mean ± SEM of three animals per group. *P <0.05, **P <0.01, ***P <0.001, and ****P <0.0001 by one-way ANOVA with Tukey’s test.

Previous studies from our group and others have determined that improvements in functional recovery are strongly correlated with enhanced myelin preservation [3,8,9,32]. To address whether XPro1595 had similar effects, we performed western blot analysis for MBP, a marker for myelin integrity, in naive and injured mice. We determined that at 7 days post-contusion there was more MBP in the XPro1595 group relative to etanercept-treated mice (Figure 5A). At 7 days after SCI, there was no difference in MBP levels between XPro1595-treated and naive controls. At 28 days post-contusion there was a significant reduction in MBP in all treatment groups compared to at 7 days after SCI and naive mice (Figure 5A). To begin exploring possible mechanisms for this, we investigated TNFR2 and TLR4, two known receptor signaling pathways implicated in positively influencing myelin expression following CNS injury or disease [3,9,44]. Using western blot analysis, we determined that TNFR2 expression remained unchanged in XPro1595-treated mice 7 days after SCI, whereas TNFR2 expression decreased significantly in both saline- and etanercept-treated mice (Figure 5B). At 28 days post-injury, TNFR2 levels were not statistically different from naive controls in any of the treatment groups. In a similar fashion, XPro1595 significantly increased TLR4 expression at 7 days after SCI compared to the other treatment groups (Figure 5C). At 28 days after SCI, TLR4 levels were significantly reduced compared to at 7 days after SCI. These data suggest that inhibiting solTNF signaling within the first 3 days after SCI promotes signaling through TNFR2 and TLR4, resulting in reduced myelin loss and significant functional recovery. GAP43 levels were not altered after SCI within the lesion site (Figure 5D).

## Discussion

In this study, we investigated the therapeutic potential of two biologic inhibitors of TNF, XPro1595 and etanercept, in a mouse model of traumatic SCI. XPro1595 is a specific inhibitor of solTNF and therefore preferentially disrupts signaling through TNFR1, without affecting tmTNF signaling through TNFR2, whereas etanercept non-specifically inhibits both solTNF and tmTNF and thus signaling through TNFR1 and TNFR2. Our results show that systemic administration of either XPro1595 or etanercept by subcutaneous injection failed to improve functional recovery and reduce tissue damage in our mouse SCI model. In contrast, central administration of XPro1595 but not etanercept significantly improved functional recovery and reduced tissue damage, as shown by smaller lesion sizes.

The findings of a lack of effect of systemically administered XPro1595 were unanticipated as we and others have previously demonstrated that systemic administration of XPro1595 significantly improved clinical scores, reduced axonal damage, and promoted remyelination in myelin oligodendrocyte glycoprotein-induced experimental EAE, a mouse model of multiple sclerosis [9,20]. The apparent discrepancy may be related to the fact that in multiple sclerosis and EAE, inflammation is a more slowly evolving sequence of events resulting from the activation of systemically circulating, peripheral immune cells and their infiltration into the CNS and subsequent attack on targets within the CNS. In multiple sclerosis, this could evolve over months to years before clinical symptoms develop, and in EAE this process usually develops within the first two weeks before clinical symptoms appear and continues to evolve throughout the course of the disease. However, in SCI, the initial and very acute inflammatory response is primarily mediated by activated cells endogenous to the spinal cord (such as microglia and astrocytes) or from the leakage of inflammatory cytokines through the disrupted blood-spinal cord barrier at the site of injury, because peripheral blood leukocytes (such as neutrophils, macrophages, and T-cells) do not arrive in significant numbers for days to weeks after the injury [2,10]. Microglia and astrocytes have the capacity to secrete and respond to numerous immunoregulatory cytokines such as TNF. For example, previous studies have shown that TNF is significantly elevated in the spinal cord within hours of injury [2,8,10,45]. This acute elevation of TNF may be toxic and hence systemically applied inhibitors may not effectively neutralize elevated levels of CNS-derived TNF before it can initiate damage. Interestingly, TNF has been demonstrated to synergize with glutamate to promote significant damage following SCI [46,47]. Therefore, together with the induced vascular reactions that might compromise the effective delivery of systemically delivered substances after the impact, it is not surprising that systemic therapies that are therapeutic in EAE may not be efficacious in traumatic SCI. Indeed, when we delivered XPro1595 or etanercept directly to the injured cord for three consecutive days and evaluated improvements in functional recovery and tissue damage, we determined that XPro1595, but not etanercept, significantly improved hind limb motor function as measured by significant improvements in BMS and rung walk tests, and reduced tissue damage. These data demonstrate that selectively inhibiting signaling of solTNF, possibly through TNFR1, is neuro/glia protective and that inhibition of signaling through both TNFR1 and TNFR2 is not protective. This further suggests that tmTNF signaling through TNFR2 is required for functional recovery and tissue repair. These data also suggest that centrally elevated levels of solTNF, rather than leukocyte-derived solTNF, is cytotoxic and should be targeted for therapy following SCI.

The role of TNF in secondary SCI is supported by numerous reports, including the observation that TNF is significantly elevated in the cord within 1 h of injury and that the anti-inflammatory cytokine IL-10 significantly improves functional recovery and reduces SCI-induced TNF [10]. This study further determined that SCI-induced monocyte/macrophage-derived TNF was not detected until 3 days post-injury and that levels in the cord were significantly elevated within 1 h following traumatic SCI, supporting our contention that elevated levels of TNF, possibly solTNF, within the spinal cord at early time points (e.g., within the first 24 h) are primarily due to synthesis by CNS-derived cells [10]. One of the mechanisms through which the acute production of solTNF is suggested to be cytotoxic is though synergistic interactions with glutamate [46,47]. These studies directly demonstrate that TNF exacerbates glutamate-mediated cell death in part through induced trafficking of GluR2-lacking α-amino-3-hydroxy-5-methyl-4-isoxazolepropionic acid receptors (AMPARs) to neuronal plasma membranes. Interestingly, TNF-induced AMPAR trafficking has been shown to occur within 1 h of SCI and to be ameliorated through TNF inhibition [47], supporting the hypothesis of involvement of CNS-derived TNF in AMPAR trafficking and pathology after SCI.

There is a rich literature discussing the involvement of TNF and other inflammatory cytokines in behaviors such as anxiety and depression [48,49], and evidence from clinical trials using anti-TNF biologic therapies for psoriasis and rheumatoid arthritis patients demonstrated that treatment caused significant changes in mood [13,50]. Elevated levels of TNF and other inflammatory mediators and their association with anxiety or depression-like behaviors are generally studied in models of acute or chronic stress; however, TNF and TNFR1 knock out mice have been shown do display a basal “anti-depressant” phenotype [51]. We reasoned that traumatic SCI is indeed a stressful stimuli and would be expected to induce anxiety-like behaviors in injured mice. In support of this hypothesis there are reports of anxiety and depression in patients with spinal cord injury [52,53]. In fact, our data show that inhibition of solTNF dramatically reduces SCI-induced anxiety-like behaviors compared to saline- and etanercept-treated mice. These data strongly suggest that inhibiting solTNF may be a viable therapeutic strategy for improving quality of life of persons suffering from chronic SCI, and that this can be achieved without compromising the patient’s innate immune response, which is the case with current anti-TNF therapies [54,55].

The improvements in functional recovery observed in our XPro1595-treated mice could be due in part to the significant reduction in tissue damage and enhanced myelin integrity, as measured by lesion volume analysis and increased MBP expression. In the present study, we observed significantly more MBP protein in Xpro1595-treated animals at 7 days post-injury but by 28 days post-injury levels had normalized between all treatment groups. Therefore, improvements in functional recovery at more chronic time intervals are likely due to a myriad of factors such as enhanced tissue preservation, reduced axonal injury, enhanced remyelination, and improved vascularization. Recent *in vivo* and *in vitro* studies have demonstrated that remyelination and neuroprotection are dependent upon signaling through TNFR2 [9,20,21,56,57]. In support of this, we observed a significant increase in TNFR2 expression in SCI mice treated with XPro1595 compared to etanercept-treated animals. Another important mediator of myelin integrity is TLR4, which has been shown to promote oligodendrocyte progenitor cell proliferation and to support oligodendrogenesis [44,58]. Our data demonstrate that there is significantly more TLR4 expression in XPro1595-treated mice. Collectively, these data suggest that modulating the local inflammatory response by inhibiting solTNF enhances myelin integrity and improves functional recovery in part through upregulation of TNFR2 and TLR4. Evidence for TNFR2 signaling as an important regulator of angiogenesis has recently been documented [59]. Using TNFR2 null mice it was demonstrated that TNFR2 signaling reduces hypoxia-mediated cell death and promotes revascularization.

## Conclusions

Inhibiting solTNF within the cord immediately following injury is therapeutic for traumatic SCI, whereas inhibiting solTNF systemically is not. This study highlights the importance of centrally-derived solTNF in the pathophysiology of traumatic SCI.

## Abbreviations

AMPARs: α-amino-3-hydroxy-5-methyl-4-isoxazolepropionic acid receptors
BMS: Basso Mouse Scale
CNS: Central nervous system
EAE: Experimental allergic encephalomyelitis
GAP43: Growth associated protein 43
GFAP: Glial fibrillary acidic protein
Iba1: Ionized calcium binding adapter molecule 1
MBP: Myelin basic protein
SCI: Spinal cord injury
solTNF: Soluble-TNF
TLR4: Toll-like Receptor 4
tmTNF: Transmembrane-TNF
TNF: Tumor necrosis factor
TNFR: Tumor necrosis factor receptor
